# Biomimetic Moth-eye Nanofabrication: Enhanced Antireflection with Superior Self-cleaning Characteristic

**DOI:** 10.1038/s41598-018-23771-y

**Published:** 2018-04-03

**Authors:** Jingyao Sun, Xiaobing Wang, Jinghua Wu, Chong Jiang, Jingjing Shen, Merideth A. Cooper, Xiuting Zheng, Ying Liu, Zhaogang Yang, Daming Wu

**Affiliations:** 1 College of Mechanical and Electrical Engineering, Beijing University of Chemical Technology, Beijing, 100029 China; 2 Department of Chemical and Biomolecular Engineering, The Ohio State University, Columbus, OH 43210 USA; 3 Tumor Marker Research Center, National Cancer Center/Cancer Hospital, Chinese Academy of Medical Sciences and Peking Union Medical College, Beijing, 100021 China; 4 School of Civil Engineering & Architecture, Taizhou University, Zhejiang, 318000 China; 5 State Key Laboratory of Organic-Inorganic Composites, Beijing, 100029 China

## Abstract

Sub-wavelength antireflection moth-eye structures were fabricated with Nickel mold using Roll-to-Plate (R2P) ultraviolet nanoimprint lithography (UV-NIL) on transparent polycarbonate (PC) substrates. Samples with well replicated patterns established an average reflection of 1.21% in the visible light range, 380 to 760 nm, at normal incidence. An excellent antireflection property of a wide range of incidence angles was shown with the average reflection below 4% at 50°. Compared with the unpatterned ultraviolet-curable resin coating, the resulting sub-wavelength moth-eye structure also exhibited increased hydrophobicity in addition to antireflection. This R2P method is especially suitable for large-area product preparation and the biomimetic moth-eye structure with multiple performances can be applied to optical devices such as display screens, solar cells, or light emitting diodes.

## Introduction

**Figure 1:**
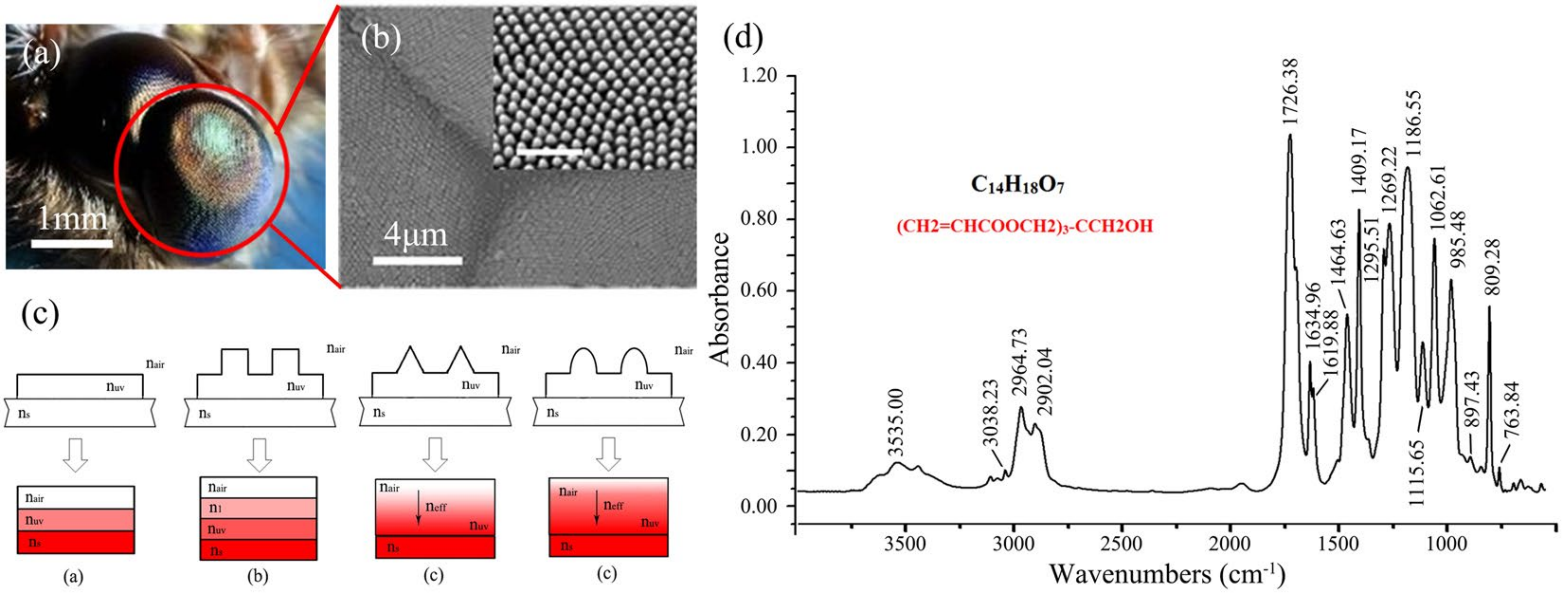
(**a**) Complete compound eyes; (**b**) SEM image of the details of ommatidia. The inset figure is magnified SEM image to show the sub-wavelength structure array in one ommatidium (The scale bar inset is 1 μm.); (**c**) Schematics of refractive index variations experienced by incident light: (i) flat UV-curable resin coating, (ii) columnar sub-wavelength structure, (iii) triangular sub-wavelength structure, and (iv) conical sub-wavelength structure; (**d**) Infrared spectrum analysis of the UV-curable resin GP756.

Research of sub-wavelength structures on the surface of moth eyes led to the original understanding of antireflective (AR) phenomenon in nature. Each ommatidium of the nocturnal moth is covered with AR nanostructures—an array of 200 to 300 nm sized pillars—which simultaneously reduce the reflection of light and enhance night vision capability. These features allow moths to see well in darkness with no reflection that could be visible to their predators^1,2^. As shown in Fig. 1a and b, the sub-wavelength structures are arranged on the surface of moth ommatidia in a highly ordered array^3^. The effective refractive index between the air and the medium of the eye changes gradually due to these AR nanostructures that are smaller than the visible wavelengths (380 to 760 nm). In this way, the incident light will not experience a sudden variation in refractive index, which would cause proportional reflections^3,4^. Following this observation of AR nanostructures, the biomimetic sub-wavelength structures also showed such an excellent antireflective property, which was known as the “moth-eye effect”^5,6^. The rapid development of nanofabrication technology in recent years, had led to the biomimetic moth-eye structures being applied to optical devices such as display screens^7^, solar cells^8,9^, and light emitting diodes (LED)^10,11^.

Until now, multilayer coating^12^ and graded index coating^13,14^ were the two most commonly used approaches to fabricate an efficient AR coating. The former is based on physical vapor deposition to obtain ultrathin inorganic layers on different substrates. However, there are several limitations to this approach. The vapor deposition method is difficult to deal with large size surfaces. Precise thickness of each inorganic layer also should be accurately designed and repeatedly optimized to ensure high transmittance in wide ranges of incidence angles and wavelengths^15^. Furthermore, multilayer coating is known to have inherent problems like absorption and scattering loss, low durability, thermal deformation, etc^16^. Inspired by moth eyes, the latter approach utilizes graded index coating, to reduce reflection of incident light. This biomimetic coating, which is made up of highly ordered sub-wavelength structure arrays, has an advantage by obtaining ultralow reflectance in wide spectrums and field ranges^17^. To date, many surface treatment methods, such as plasma etching, electron-beam lithography, sol-gel method, and self-assembly method, have been developed and utilized to fabricate antireflection sub-wavelength structures on different kinds of substrates^3,18–20^. With benefits including its high precision, high efficiency, operation simplicity and low cost, the nanoimprint lithography (NIL) method^16,21,22^, which includes thermal nanoimprint lithography and ultraviolet nanoimprint lithography (UV-NIL), is also regarded as one of the most promising techniques for antireflection.

NIL is one of the most reliable and effective techniques for the fabrication of nanosized structures at a reasonably low cost^23^. This technique was firstly proposed by Chou *et al*.^24^ in 1995 for the preparation of sub-25 nm structures on polymer surfaces. Then in 1999, Grant Wilson and co-workers extended the NIL technique to nanostructure fabrication using UV-curable resins^25^. Therefore, thermal and UV imprinting are involved in the NIL technique, and both can be applied directly to polymer surfaces. In NIL process, a patterned mold (typically composed of Silicon, Ni metal, and polymer substrates) was covered with a layer of UV-curable resin or thermoplastic polymer and imprinted by applying appropriate imprinting force and temperature for a reasonable period.

In the last decade, NIL technique had been intensively used in the fabrication of AR products. Jung *et al*.^26^ transferred AR moth-eye structure onto the surface of glass substrates using UV-NIL method. The normal incident transmittances increased from ~91% for a bare glass substrate to ~93% and ~97% for single and double side patterned AR products over a 400 to 800 nm wavelength range. Kim and co-workers^16^ reported a similar research work based on thermal-NIL process on PC films with an average normal incident transmittance of ~92% (400 to 800 nm wavelength range). Abbott *et al*.^27^ reported a soft roller NIL technique for the preparation of subwavelength silicon moth-eye structures. Their products presented an average reflection of 3% in the spectral range of 400–1000 nm for 45° incident angle. Besides enhanced AR performances, advantageous hydrophobic behavior is a common by-product of the sub-wavelength moth-eye structure^28,29^. Normally, the water contact angles of AR structured samples prepared by NIL techniques on polymer or glass substrates can reach the range from ~100 to ~120° with different geometric structures^3,26^. For the AR structures formed by some chemical process (e.g. reactive ion etching) or special hierarchical structures, the AR products can present superhydrophobic performance with water contact angles higher than 150° (even higher than 170°)^21,30^.

Based on the effective medium theory^31^, the sub-wavelength structures will behave as an effective medium when incident lights interact with the coating surface. For example, look at the case of UV-curable resin. The schematics of the refractive index variations are shown in Fig. 1c. The incident lights interacting with single-layer coating with no AR structure (Fig. 1c-i) will experience three different refractive indexes, the refractive index of air (n_air_), UV-curable resin (n_uv_), and substrate (n_s_), respectively. However, a coating with columnar sub-wavelength structure arrays (Fig. 1c-ii) would have an additional effective refractive index (n_1_), which is controlled by the ratio between the structures and channels. While the effective refractive index (n_eff_) of the conical subwavelength structure with triangular profile (Fig. 1c-iii), which is equal to infinite thin layers with linearly changed duty ratios, will experience a linear variation from n_air_ to n_uv_. The schematic of biomimetic moth-eye structure, which is the focal point of this work, is presented in Fig. 1c-iv. As a variant of the conical subwavelength structure, the n_eff_ of this moth-eye structure with parabola-shaped profiles would experience a gradual variation instead of a linear one^3,32^.

In this study, sub-wavelength anti-reflection structures were fabricated by the Roll-to-Plate (R2P) UV-NIL method on the surface of transparent polycarbonate (PC) substrate. A GP756 UV-curable resin provided by Everwide Chemical Co., Ltd. was chosen for the preparation of AR coatings. The main component of GP756 was found to be Pentaerythritol Triacrylate (PETA) using infrared spectrum analysis (as shown in Fig. 1d). The resulting products showed both AR and hydrophobic behavior. The R2P UV-NIL method is especially suitable for large-area products preparation and its efficiency is very high—the main procedure can be accomplished within 1 or 2 minutes. Furthermore, the cost for R2P UV-NIL method is much lower than traditional techniques like etching and electron-beam lithography. Therefore, the proposed fabrication method and the fabricated biomimetic moth-eye products with multiple performances could be potentially applied to optical devices such as display screens, solar cells, and LEDs in the near future.

## Results and Discussion

### SEM image of PDMS flexible female mold

**Figure 2:**
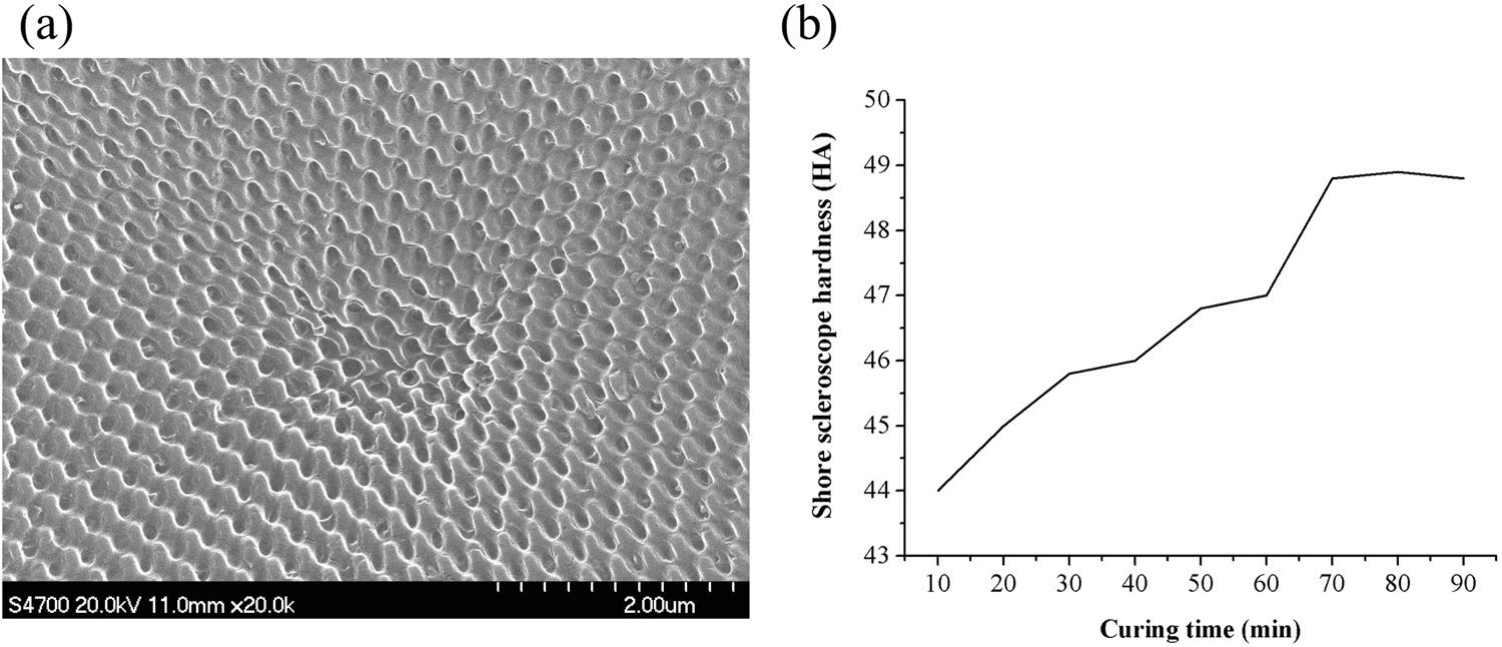
(**a**) SEM image of PDMS flexible female mold; (**b**) Shore scleroscope hardness variation of PDMS mold with different curing time under 110 °C.

Figure 2a shows a typical SEM image of the PDMS flexible female mold fabricated by the replication of the Ni mold. AR nanostructures with an average cycle of ~250 nm can be obtained. The PDMS kit consisted of a silicon elastomer base and a curing agent; a ratio of 10 to 1 was used to fabricate the PDMS flexible female mold in this paper. Among the fabrication procedures of the sub-wavelength structured surface, the preparation of the PDMS flexible female mold was an optional process. As the duty ratio of the sub-wavelength structure used in this paper was 0.5, the pattern on the female and original molds are the same. However, the application of PDMS mold is a double-edged sword. On the one hand, the application of PDMS flexible female molds can greatly extend the service life of the original Ni mold with less pollutions and damages. On the other hand, more operation steps in the whole fabrication process of AR sample would greatly raise the defect rate, since more monitoring and controlling of unfavorable factors (e.g. incomplete filling, demolding defects, etc.) are needed.

When the PDMS mold was utilized, it is important to ensure the geometry stability of PDMS flexible female mold under specific imprinting force. That is, the PDMS mold should be strong enough to avoid structure disordered in AR samples. In order to get better AR nanostructures with UV-curable resin, PDMS molds with higher hardness were preferred. Thus, a simple experiment of hardness testing was performed to seek better curing parameters of PDMS (as show in Fig. 2b). The geometry of nanostructures would change under a variety of conditions, especially the curing temperature and curing time^33,34^. The hardness of cured PDMS will increase almost linearly with an increase in curing temperature and curing time until the upper limit is reached. The variation of Shore scleroscope hardness of PDMS with different curing time under 110 °C is shown in Fig. 2b. All the shore hardness results were an average value of five different samples. It can be seen that the Shore scleroscope hardness of cured PDMS gradually increased from 44.0 HA to 48.8 HA, while the curing time increased from 10 to 70 min, and basically remain unchanged even if the curing process lasted 90 min. This means that PDMS was fully cured after 70 min and no marked changes would occur after that. Therefore, we selected 70 min as a standard for the curing process of the PDMS flexible female mold.

### Morphology and reflectance of AR sub-wavelength structured surfaces prepared at different imprinting forces

The stage moving speed is an important parameter of the UV-NIL process which should be carefully determined. On the one hand, it should be slow enough to permit sufficient time for resin filling and curing. On the other hand, the stage moving speed should be fast enough to avoid over curing and related collapsing defects. Thus, a stage moving speed of 3 mm/s was chosen for all the experiments discussed in this paper.

**Figure 3:**
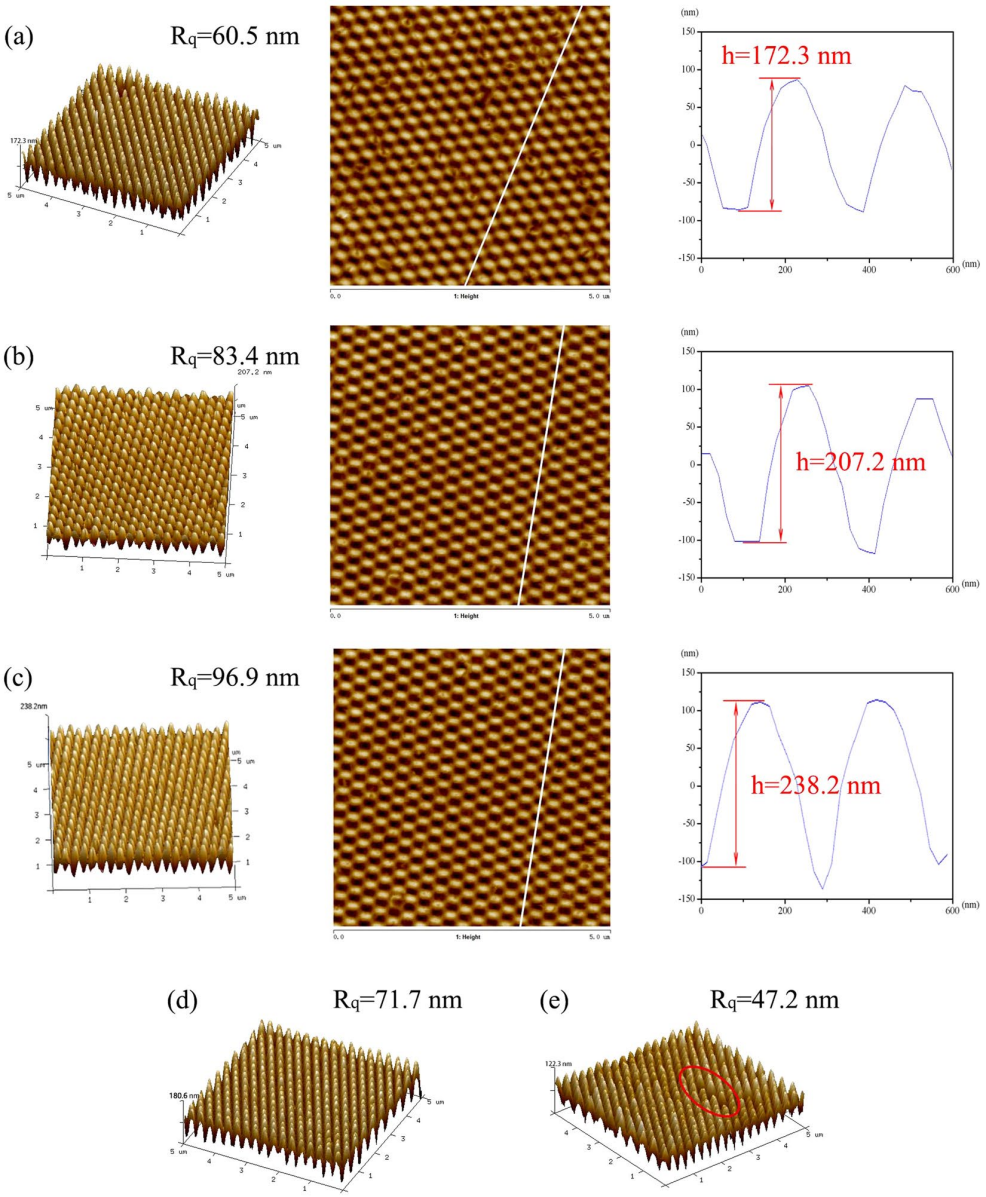
(**a–c**) AFM images of AR sub-wavelength structures fabricated using ultraviolet nanoimprint lithography process with an imprinting force of: (**a**) 150 N, (**b**) 250 N, and (**c**) 350 N (Typical three-dimensional image (left), two-dimensional top view image (middle), and line profile image (right) using AFM); (**d**,**e**) Three-dimensional AFM images of AR sub-wavelength structures fabricated using UV-curable resins with different viscosities of: (**d**) 400 cps and (**e**) 800 cps.

Figure 3a–c shows the top view and three-dimensional AFM images of AR sub-wavelength structure arrays prepared at different imprinting forces, which were 5 × 5 μm in area. The line profile images for selected typical parts of these samples are also presented. Figure 3a,b and c correspond to imprinting forces of 150 N, 250 N and 350 N, while the viscosity of UV-curable resin remained constant at 100 cps. It can be seen from Fig. 3 that the typical heights of AR sub-wavelength structures increased along with the increasing imprinting forces. While the imprinting force went up from 150 N to 350 N, the typical height increased from 172.3 nm to 207 nm to 238.2 nm. As the pattern height of the AR sub-wavelength structure on Ni mold was 250 nm, a height of 238.2 nm obtained with an imprinting force of 350 N and a viscosity of 100 cps was already quite close. Continuing to increase the imprinting force to the maximum value of 500 N would not lead to an increased height. Actually, it was almost theoretically impossible to get a replication rate of 100% on account of the slightly shrinking UV-curable resin during the curing process as well as probable defects during the demolding process. The typical heights of the AR sub-wavelength structures prepared with the PDMS flexible female mold will decrease a little more because of defects due to the deformation of PDMS under high pressure.

As can be seen from the line profile images in Fig. 3, all the AR sub-wavelength structures obtained under different imprinting forces were parabolic in shape. According to effective medium theory, the parabola-shape profiles can result in a nearly linear refractive index gradient, which will lead to a sufficient reduction in surface reflection of the AR products.

**Figure 4:**
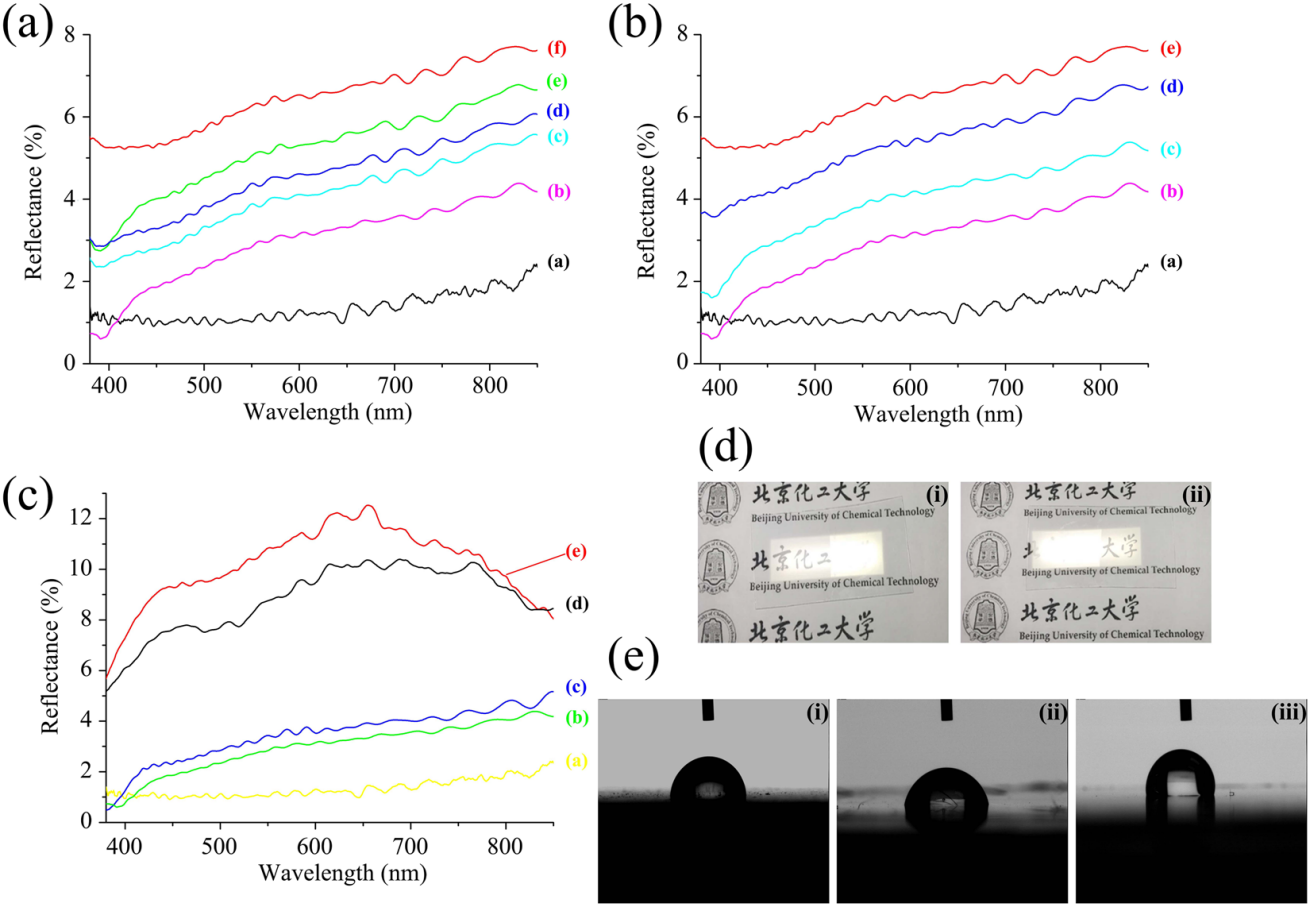
(**a**) Optical reflectance spectra of AR sub-wavelength structured samples. (Curves a-e represent imprinting forces of 350, 250, 200, 150 and 100 N; curve f represents the flat UV-curable resin coated PC substrate); (**b**) Optical reflectance spectra of samples with different parameters (imprinting force and resin viscosity). (Curves a-d represent parameters of (**a**) 350 N, 100 cps; (**b**) 250 N, 100 cps; **(c**) 250 N, 400 cps; and (**d**) 250 N, 800 cps, curve e represents the flat UV-curable resin coated PC substrate); (**c**) Measured angle-dependent reflectance at different incidence angles. (Curves a-e represent incidence angles of 0°, 30°, 50°, 60°, and 75°); (**d**) Photographs of half side AR sub-wavelength structured samples exposed to conventional room illumination. Characters are visible only under the (i) left and (ii) right half, since they are cover with AR structures; (**e**) Water contact angles of (i) bare PC substrate, (ii) flat UV-curable resin coated PC substrate, and (iii) AR sub-wavelength structured sample fabricated with an imprinting force of 350 N and a resin viscosity of 100 cps (sample 4 in Table 1).

Figure 4a shows the optical reflectance spectra at normal incidence of flat UV-curable resin coated PC substrate and AR sub-wavelength structured samples fabricated using the UV-NIL process with different imprinting forces. The average reflectances of the AR sub-wavelength structured samples were 1.21, 2.71, 3.75, 4.25, and 4.90%, for imprinting forces of 350, 250, 200, 150, and 100 N, respectively (calculated from the data ranging from 380 to 760 nm of curve (a) to curve (e) in Fig. 4a). All these samples mentioned above were less reflective than flat UV-curable resin coated PC substrate, which was 6.22% in average based on the data of curve (f). It can be concluded from the results of Figs 3 and 4a that the reflectance decreases with increased heights of the AR sub-wavelength structures.

### Morphology and reflectance of AR sub-wavelength structured surfaces prepared using UV-curable resins with different viscosities

In this section, the influence of resin viscosity on AR sub-wavelength structures was further researched. UV-curable resins with different viscosities of 100 cps, 400 cps and 800 cps were prepared, while the imprinting force was kept constant at 250 N during the whole UV-NIL process. Using the same analysis methods in section 3.2, morphology and reflection performances were measured to evaluate the AR sub-wavelength structured samples.

Figure 3d and e show the three-dimensional AFM images of AR sub-wavelength structures prepared using UV-curable resins with viscosities of 400 cps and 800 cps, respectively. Meanwhile, the morphology of samples fabricated with a resin viscosity of 100 cps and an imprinting force of 250 N have already been presented in Fig. 3b. Contrary to the relationship between imprinting force and typical height, a declining trend between resin viscosity and typical height is observed. With different resin viscosities of 100, 400, and 800 cps, the typical heights of AR structures decreased from 207.2 nm to 180.6 nm and then sharply declined to 122.3 nm. Along with the increase of resin viscosity came an increase in imprinting defects. As shown in the red circle area of Fig. 3e, several cycles of the AR sub-wavelength structure disappeared, while the AR structure array in Fig. 3b and d were far more uniform. Therefore, lower resin viscosity and reasonable higher imprinting force were conducive to higher replication rate in the UV-NIL process.

Besides, the demolding process would greatly influence the final shape of AR structures. In order to get well shaped AR structures, the following means were executed. Firstly, silanization treatment had been performed to the Ni mold to avoid pollution by UV-curable resin during imprinting. Secondly, optional Ar Plasma treatment to Ni mold and O_2_ plasma to PC substrate can made them more hydrophobic and hydrophilic, respectively. Leading to stronger binding between PC substrate and UV-curable resin. Thirdly, the chosen resin (GP756) could be used as polymer adhesives, which meant GP756 had much stronger adhesive force with polymers than metals. All of these above made the AR structures formed from UV-curable resins tended to bind on the surface of PC substrate during demolding. Furthermore, the operation of demolding process should begin from one side slightly to avoid demolding defect caused by stress concentration.

A further comparison of the optical reflectance spectra at normal incidence is shown in Fig. 4b. Curve (a) and curve (b) in Fig. 4b match those in Fig. 4a, while curve (e) in Fig. 4b and curve (f) in Fig. 4a also represent the reflectance result of the same sample (flat UV-curable resin coated PC substrate). In order to exhibit the difference in reflectance property caused by resin viscosity variation, the data was listed together with the samples fabricated using UV-curable resin with viscosities of 400 cps (curve (c)) and 800 cps (curve (d)). It can be seen that along with the increase of resin viscosity, the corresponding reflectance increased from 2.71% to 3.71% and finally reached 5.49% with a resin viscosity of 800 cps (calculated from the data ranging from 380 to 760 nm). Thus, these results led to a conclusion the same as the previous section; the reflectance would decrease with increased heights of AR sub-wavelength structure.

The morphology and reflectance results of several representative AR sub-wavelength structured samples mentioned in section 3.2 and 3.3 are listed in Table 1 for a better comparison. Sample 1 was the flat UV-curable resin coated PC substrate, while sample 2 through 6 were fabricated using UV-NIL process with different parameters. Among all the samples listed in Table 1, sample 4 got the highest replication rate at 95.28% and best reflection performance at 1.21% with an imprinting force of 350 N and a resin viscosity of 100 cps. Either lower imprinting force or higher resin viscosity would cause a decline in both morphology and reflection performances of AR sub-wavelength structured samples. That is to say, higher heights of AR sub-wavelength structures, which can be obtained with lower resin viscosity and reasonable higher imprinting force during UV-NIL process, are conductive to better reflection performance.

**Table 1:** Comparison of the morphology and reflectance results of flat UV-curable resin coated PC substrate and several representative AR sub-wavelength structured samples.

Sample Number	Processing Parameters		Performances			
	Imprinting Force (N)	Resin Viscosity (cps)	Average Height (nm)	Root Mean Square Roughness/R_q_(nm)	Replication Rate (%)	Reflectance at Normal Incidence (%)
1	/	/	/	/	/	6.22
2	150	100	172.3	60.5	68.92	4.25
3	250	100	207.2	83.4	82.88	2.71
4	350	100	238.2	96.9	95.28	1.21
5	250	400	180.6	71.7	72.24	3.71
6	250	800	122.3	47.2	48.92	5.49

Although the reflection performance of an AR sub-wavelength structure can be significantly affected by the variation of average height or replication rate, it was still lower than that of flat UV-curable resin coated PC substrate. This proved that the biomimetic moth-eye structure fabricated using UV-NIL process actually had a positive effect as an antireflection coating.

### The influence of incidence angle on the reflectance of AR sub-wavelength structured surface

Sample 4 in Table 1, which had the highest replication rate and best reflection performance, was selected for further research on the angle-dependent reflection performance of AR sub-wavelength structures fabricated using UV-NIL process. The influence of incidence angle on reflectance was characterized with results shown in Fig. 4c. Although the average reflectance was clearly enhanced with the increase in incidence angles, it remained below 4% at 50°. After that, further promotion of incidence angle leads to a steep increase in reflectance to around 10%. Based on the reflection data ranging from 380 to 760 nm of curve (a) to (e), the average reflectance at 0°, 30°, 50°, 60°, and 75° was 1.21%, 2.70%, 3.20%, 8.76%, and 10.40%, respectively.

All the results listed above demonstrate that the light propagating on AR sub-wavelength structures produces a continuous variation in the refractive index between the air and the medium. As shown in Fig. 4d, the reflected lighted was effectively decreased by AR sub-wavelength structures. Figure 4d presents two samples exposed to conventional room illumination, in which AR sub-wavelength structures were on the left (Fig. 4d-i) and right (Fig. 4d-ii) side, respectively. The Chinese characters under the AR structures were relatively unambiguous, while those under bare PC substrates can’t be distinguished completely. As the incandescent lamp was in the middle of the lampshade, the light intensity in this area was much higher and made the characters in this position even harder to distinguish. The results can be explained as follow. A certain amount of lamplight would be reflected when they reached the surface of bare PC substrates. The influence of reflected lamplight is quite considerable and made the characters under it invisible to human eyes even though the bare PC substrate was transparent. In case of PC substrate covered with AR sub-wavelength structures, the amount of reflected lamplight will be greatly reduced, which made the characters visible even under intense light.

The AR samples mentioned in this study were coated with the UV-curable resin sub-wavelength structures on only one side (on the top). It should be pointed out that the optical reflectance can be further reduced with AR sub-wavelength structures on both sides (on the top and bottom) of the PC substrate^35^. In our R2P UV-NIL process, the application of a PDMS flexible female mold was provided as an optional method to fabricate AR sub-wavelength structures on a polymer substrate. However, the PDMS flexible female mold itself also had the potential to be applied as soft AR products^36,37^.

### Hydrophobic performance of AR sub-wavelength structured surface

Nanostructure surfaces with large roughness can enhance the hydrophobic performance^38,39^, which is very useful for outdoor applications. As a biomimetic moth-eye structured surface, the AR sub-wavelength structures fabricated using UV-NIL process can induce self-cleaning of small particles and dusts on sample surfaces^30,40^. The photographs of water contact angle on the surface of a bare PC substrate, a flat UV-curable resin coated PC substrate, and an AR sub-wavelength structured sample fabricated using UV-NIL process with an imprinting force of 350 N and a resin viscosity of 100 cps are shown in Fig. 4e. As can be seen from these figures, the corresponding water contact angles were 86.7°, 80.7°, and 104.2°, respectively. Only the AR sub-wavelength structure sample exhibited hydrophobic performance with a contact angle higher than 90°. The increase of water contact angle can be attributed to surface roughness increasing and water contact area decreasing caused by AR sub-wavelength structures. Increasing hydrophobic performance also can make it easier for water droplets to move around on the surface, which means the dusts on our AR sub-wavelength structured samples can be washed off much more easily^41,42^. Furthermore, once the AR product was applied as the cover layer of solar cells, the install inclination angle of solar cell panels (~40° in the middle and lower reaches of Yangtze River in China) would make it easier for the self-cleaning of dust deposition with the help of dew and rainwater.

In the present work, Roll-to-Plate (R2P) ultraviolet nanoimprint lithography (UV-NIL) method was introduced for the fabrication of antireflection (AR) sub-wavelength structures. This technique was especially suitable for the continuous preparation of large-area AR products. The height of periodic sub-wavelength structures with parabola-shaped profile can be controlled by the variation of processing parameters, such as stage moving speed, imprinting force and resin viscosity, during UV-NIL process. It will also affect the change of optical reflection performance of obtained AR samples. In this study, the influence of imprinting force and resin viscosity were researched and discussed. An imprinting force of 350 N and a resin viscosity of 100 cps were found to be the best parameters, and the average reflectance of this AR sub-wavelength structured sample was only 1.21% in the visible light range at normal incidence. Furthermore, we found the biomimetic moth-eye structured samples also presented hydrophobicity and a water contact angle of 104.2° was obtained. The biomimetic moth-eye AR structured surfaces with dual functionality can be extended to future applications in the fields of display screens, solar cells, light emitting diodes, and related optical devices.

## Methods

### Experimental device and mold

**Figure 5:**
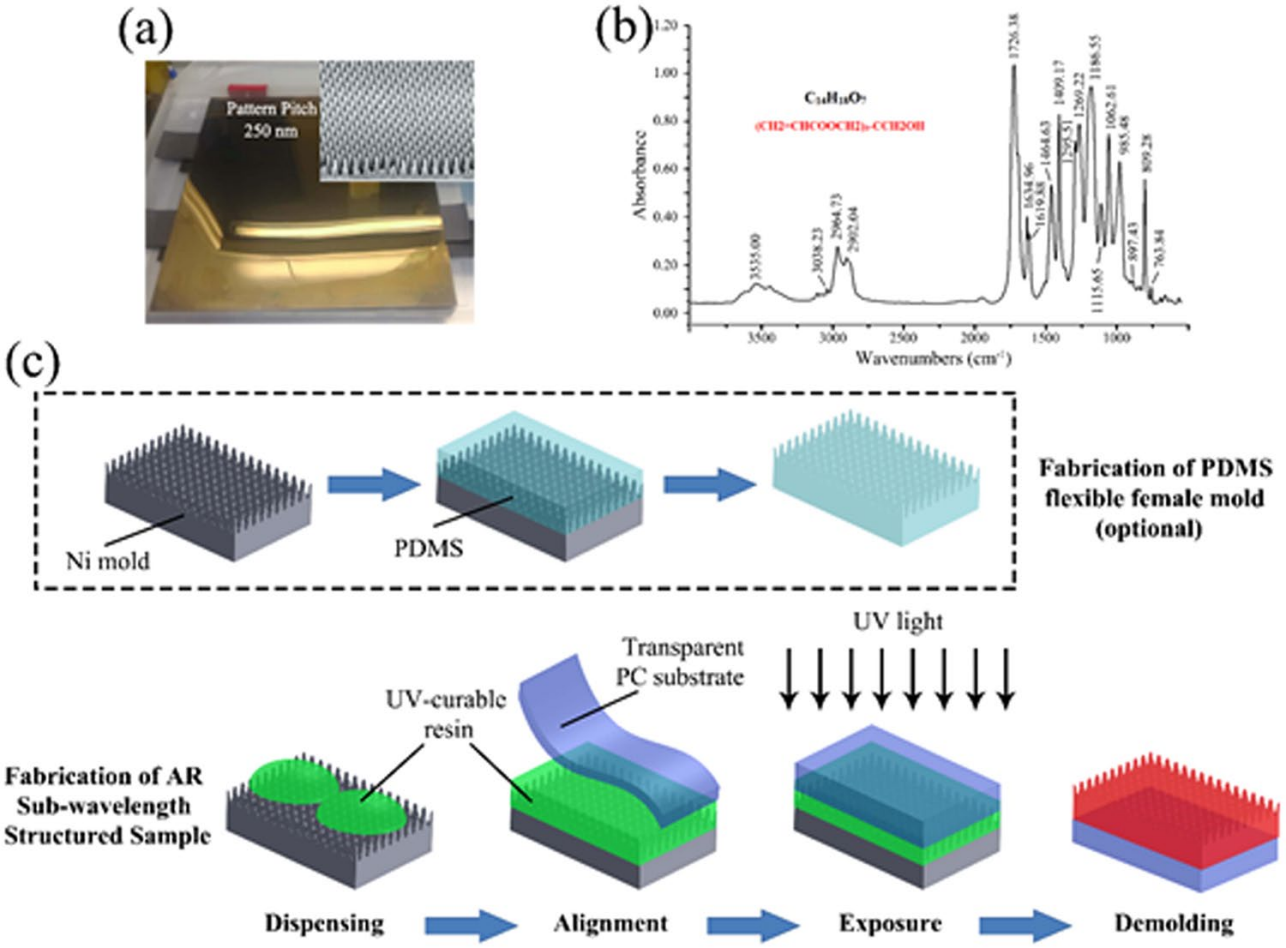
(**a**) Details of Ni mold on macro and nano scale; (**b**) Infrared spectrum analysis of the UV-curable resin GP756; (**c**) Schematic for the fabrication procedures of AR sub-wavelength structured sample.

A R2P UV Roller Press Scan imprinter of ERP-210 (Engineering System Co., Ltd., Japan) was used to fabricate AR sub-wavelength structures. As shown in Fig. 5a, the experimental device was composed of five parts: the roller part, the stage part, the UV light, the force control, and the operational part. The roller (made of urethane rubber with A50 shore hardness) was driven with an air-pressurized cylinder and could provide a pressing force up to 400 N. UV LED lights with a wavelength of 365 ± 5 nm and an intensity of 20 mW/cm^2^ were right under the roller. The effective imprint area of ERP-210 was 180 × 250 mm.

The Ni mold with the AR sub-wavelength structure was provided by KYODO International Co., Ltd., Japan. The sub-wavelength structure was made by the method of two-beam laser interference photolithography, and the design of the mold was mainly focus on the mimic of moth-eye structures. Figure 5b and the inset image showed the details of the Ni mold on both macro and nano scale. The duty ratio of the sub-wavelength structure was 0.5, while the pattern had a height of 250 nm and a diameter of 285 nm. The overall size of the Ni mold was 180 × 200 mm. After the experiments were complete, isopropyl alcohol (IPA) at 99% concentration was used as mold cleaning agent.

### Materials

A kit (containing silicone elastomer base and curing agent) for a poly(dimethylsiloxane) (PDMS) prepolymer of SYLGAR^®^ 184 (Dow Corning Corp.) was used for flexible female mold replication. Transparent PC substrates with a thickness of 0.25 mm were purchased from Dongguan Lingmei New Materials Co., Ltd., and its average transmittance was above 89%. UV-curable resin with a viscosity of 800 cps at 25 °C (GP756, Everwide Chemical Co., Ltd., Taiwan) was used for preparing the AR coatings. GP756 was a radical curing resin with high suitability and hardness, which was suitable for the fabrication of nanostructures with high aspect ratio. The GP756 resin coating on PC substrate had a shore hardness of D86, while the pencil hardness of the cured resin film was 4 H. Thus, the imprinted AR structure presented relatively high mechanical stability and durability. The AR performance of products still maintained after flushed with water and scrubbed with Kimwipe paper for more than 100 cycles. The result of infrared spectrum analysis of GP756 is shown in Fig. 1d. The graph shows that the main component of GP756 was Pentaerythritol Triacrylate (PETA).

### The dilution of UV-curable resin

In this paper, the influence of viscosity on the processing effect of an AR sub-wavelength structure was discussed. In order to get UV-curable resin with lower viscosity, Methoxy Polyethylene Glycol 400 Methacrylate (MPEG400MA) with a viscosity of 23 cps at 25 °C (Guangzhou Deco Composite Technology Co., Ltd., China) was chosen as the diluting agent. With the help of MPEG400MA, UV-curable resin with different viscosities of 100 cps, 400 cps and 800 cps were prepared by mixing in the correct proportions.

### Preparation of sub-wavelength structured surfaces

Figure 5c shows the schematic for the fabrication procedures of the AR sub-wavelength structure surface using the R2P UV-NIL process. First, a PDMS prepolymer layer was prepared by casting on the surface of the Ni mold at room temperature. The flexible female mold with an opposite structure from the Ni mold can be achieved after heat curing for 70 minutes at 110 °C and demolding (optional). Secondly, an ordered UV-curable resin droplet array was patterned onto the PDMS flexible female mold (or the original Ni mold) through an automatic three-dimensional dispensing system (YSD-331-X, Shenzhen Yuanshang Automation Technology Co., Ltd., China), then, a transparent PC substrate was laid on top. After that, the R2P UV-NIL process was performed under UV light conditions for 2or more cycles with a stage moving speed ranging from 1 mm/s to 5 mm/s using PDMS female mold (or original Ni mold). An imprinting force ranged from 50 N to 500 N during the whole cycles mentioned above at room temperature. Finally, the AR sub-wavelength structured biomimetic moth-eye product was obtained after the curing and demolding processes.

### Samples characterizations

The morphology of AR sub-wavelength structured surfaces was characterized using a field emission scanning electron microscope (FE-SEM, S-4700, Hitachi, Japan)^43^ and an atomic force microscope (AFM, Dimension FastScan, Bruker, Germany). The reflectance of AR sub-wavelength structured surface at normal incidence was observed by a benchtop spectrophotometer (Color i5, X-Rite, America). The instrument was equipped with a pulsed xenon arc lamp (artificial daylight 6500 K, D65 calibrated). The reflectance spectra at different angles (30°, 50°, 60° and 75°) were observed using a variable angle multifunctional optical characteristic measuring system. A high-resolution optical fiber spectrometer (AvaSpec-ULS364-USB2, Avantes, Holland) was used to transmit the spectral data to a computer, and the AvaSoft-Full software was further applied for data processing and recording. The water contact angles of bare PC substrate, flat UV-curable resin coated PC substrate and the AR sub-wavelength structured sample were measured a drop shape analyzer (KRUSS DSA100) to evaluate their hydrophobic performance.
